# Adoption of a Drug Adherence App to Improve Medication Adherence: A Randomized Controlled Trial

**DOI:** 10.1002/hsr2.71729

**Published:** 2026-01-20

**Authors:** Martin C. S. Wong, Claire Chenwen Zhong, Siu Hin Wong, Chung Yi Lo, Man Kin Yim, Junjie Huang

**Affiliations:** ^1^ Jockey Club School of Public Health and Primary Care, Faculty of Medicine The Chinese University of Hong Kong Shatin Hong Kong SAR China; ^2^ Centre for Health Education and Health Promotion, Faculty of Medicine The Chinese University of Hong Kong Shatin Hong Kong SAR China; ^3^ The School of Public Health Peking University Haidian Beijing China; ^4^ The School of Public Health The Chinese Academy of Medical Sciences and The Peking Union Medical Colleges Dongcheng District Beijing China; ^5^ The School of Public Health Fudan University Yangpu District Shanghai China

**Keywords:** blood pressure, hypertensive, MMAS‐8 score, randomized controlled trial

## Abstract

**Background and Aims:**

The “My eDrug Manager” app provides Hong Kong patients with detailed medication guidance and reminders, but its impact on medication adherence and blood pressure control among older adults with hypertension is not well‐studied. This study aims to assess the impact of the “My eDrug Manager” mobile app on medication adherence and blood pressure control in hypertensive older adults in Hong Kong.

**Methods:**

This randomized controlled trial included 569 older adults who were on antihypertensive medication, possessed a smartphone, but demonstrated poor medication adherence. Participants were randomly assigned to either the intervention group, which used the “My eDrug Manager” app and received baseline instructions and adherence pamphlets, or the control group, which received standard care instructions and the same pamphlet. Adherence was measured using the Morisky Medication Adherence Scale (MMAS‐8), with data collected at 3, 6, and 12 months. Statistical comparisons between groups were conducted using t‐tests and chi‐square tests.

**Results:**

At 12 months, the intervention group had a significantly higher mean MMAS‐8 score (7.06 ± 1.40) compared to the control group (6.56 ± 1.44, *p* < 0.001). The proportion of participants with optimal adherence was also higher in the intervention group (68.6% vs. 57.6%, *p* =0.007). However, no significant differences in blood pressure control were observed between the groups.

**Conclusion:**

The application demonstrates the potential to improve medication adherence among older adults with hypertension, highlighting the need for integrating digital tools into care programs while also pursuing additional strategies for better blood pressure control.

## 1 Introduction

Medication adherence is the degree to which a person's drug‐taking habits align with the prescribed dosages from medical professionals. Since adherence has an impact on medication effectiveness and the management of chronic illnesses, it is crucial [1]. The World Health Organisation (WHO) has chosen “adherence enhancing” as a tactic to successfully address chronic illnesses. On the other hand, non‐adherence describes actions that deviate from doctor's orders, such as inappropriate ingestion, overuse, and underuse [2]. Among the senior population, drug safety, hospitalisations, and healthcare expenses are becoming more significant public health issues. It has been estimated that in the older adults population, inadequate adherence to medicine accounted for about 25% of avoidable adverse drug reactions (ADRs) [3, 4]. As for the drug‐related hospitalizations that occurred in the United States, between 33% and 69% are attributable to suboptimal medication adherence [3]. Each year, poor medication adherence induces an avoidable healthcare cost that ranges between USD 100 billion and USD 300 billion in the United States. Non‐adherence is therefore one of the major public health issues imposing a significant financial burden on the healthcare systems on a global basis. Poor adherence was also a source of frustration and job dissatisfaction among health professionals where medical services are delivered [5].

Medical costs, the ageing population, and the prevalence of adverse drug reactions (ADRs) among older adults with multiple morbidities are all on the rise in Hong Kong. The rising rate of polypharmacy and poor rate of medication adherence among the older adults represent new health issues that must be properly controlled using an inventive tool. The first comprehensive, user‐friendly medication management app created by chemists for the Hong Kong public is called “My eDrug Manager.” It gives patients medication monographs authored by licensed chemists, reminding them to take the recommended dosage at the recommended time. The elements of “My eDrug Manager” are acknowledged as an evidence‐based treatment for individuals with long‐term conditions that are often utilised in Western nations [5]. Therefore, it should be useful in improving senior hypertensive patients' medication adherence. It has several key features: (1). “Drug Info”— provides users with easy to read and concise information about their drugs, including their nature, indication, side effects, and precautions, and so forth. (2). “My Drugs”—builds the own medication profile of users to inform their doctor, pharmacist or other healthcare professionals; the patient only needs to show them if needed; it allows them to manage their stock and reminds them before the stock runs out. (3). “Schedule”—sets the dose and time and it will remind users to take the correct amount at the right time. (4). “Pill Identifier”—provides users with many default shapes, single or multi colours, patterns of drugs, and allows search for text markings. (5). “Drug News”—updates the users with the latest news announcements about medicines by the Drug Office, Department of Health, HKSAR Government with hyperlink to the news website. However, there is a dearth of study in Hong Kong regarding the efficacy of this application and how users see it. By conducting a randomized controlled study, we aimed to improve the level of medication adherence and examine the optimal blood pressure control among hypertensive population via the use of “My eDrug Manager” as a patient empowerment tool.

## 2 Methods

### 2.1 Ethics Approval Statement

This study was approved by the Joint Chinese University of Hong Kong—New Territories East Cluster (CUHK‐NTEC) Clinical Research Ethics Committee; reference no. 2018.650, Hong Kong SAR. Written consent was obtained from participants.

#### 2.1.1 Trial Design

Previous study revealed a mean age of 45.4 years in a study population of 4800 hypertensive participants [6]. Our study adapted a more flexible inclusion criteria of aged 40 years or older accounted for the error, standard deviation and cultural difference between different study populations. Potential participants were directed to relevant parties for eligibility assessment and provided with informed consent. Eligible participants then completed a baseline questionnaire on socio‐demographics; past medical history; information on drug prescription and their overall level of adherence with their prescriptions as measured by the MMAS‐8.

### 2.2 Study Participants

A total of 569 community‐dwelling older adults subjects aged 40 years or older were recruited based on the following criteria: (i) currently taking at least one antihypertensive medication prescribed by their primary care physicians; (ii) assessed with poor medication adherence (Morisky Medication Assessment Scale, MMAS‐8 ≤ 6); (iii) possessing a mobile phone without any existing adherence applications; (iv) no medical conditions that prevent them from providing informed consent or understanding the programme content; and (v) not living with caregivers who regularly remind them on their medication taking schedule.

### 2.3 Interventions and Controls

Out of the total participants, 290 participants were randomised to the intervention arm through a computer‐generated randomization process using the SCRAED software. The intervention group received the eDrug Manager mobile app installed on their phones, with training provided at baseline to ensure proper usage. Participants were required to bring their smartphones and medications to the training. The team of trainers offered standardized services to the participants on the usage of the app, where the content and format of the workshops to be delivered. The training class lasted for 45 minutes to 1 hours. Responsible staff assisted the subjects to install the app and teach them how to use the different functions in detail via “one‐on‐one coaching”. 40–50 training class with 16–20 subjects each class to deliver training on the usage of the app for the participants. During the class, our project coordinator shared the link to download My eDrug manager, helping subjects to install via a one‐to‐one coaching, introduced and taught subjects to use each function of focal app, and ensure that they scheduled their medication correctly in the app. In addition to app installations, participants of the intervention group also received pamphlets as a reminder of the importance of medication adherence.

Participants with Blood pressure (BP) ≥ 180/110 mmHg were advised to consult their family doctors. Another 279 participants were randomized to the usual care group, which received standard care based on patient care guidelines, along with the same pamphlet on medication adherence provided at baseline.

### 2.4 Measurement of Outcomes

The effectiveness of the mobile application was evaluated using BP and the MMAS‐8 as primary outcomes, measured at baseline, 3, 6, and 12 months. Blood pressure measurement were done by using an automated sphygmomanometer (ALPK2DS‐182) in the right arm with an appropriate cuff size. The measurements were taken after the participants resting in a sitting position for 5 minutes, at least 1 hour after the subject's last meal, and at least 30 minutes after smoking or consuming caffeinated beverages. This method of measuring blood pressure followed the same procedure in previous published study [7]. The machine and method of measurement were consistent in all assessment time points to reduce bias. BP measurements were conducted by trained staff to ensure consistency, while medication adherence was assessed using the MMAS‐8. The MMAS‐8 is a validated tool for measuring adherence in hypertensive patients, with strong correlations to BP control. Scores range from 0 to 8, with an alpha coefficient of 0.83, demonstrating good reliability [8, 9, 10], and had higher reliability than other self‐reported adherence scale for medication adherence in patient, such as MARS‐5 (Medication Adherence Report Scale) (Cronbach's *α*: 0.75) and MMAS‐4 (Morisky Medication Adherence Scale 4) (Cronbach's *α*: 0.61) [11]. Furthermore, MMAS‐8 consisted more details item than MMAS‐4 to evaluate the medication adherence in patients [12]. Its sensitivity to identify high versus low adherers was shown to be 93% when a cut‐off of 6 was used [9]. The MMAS‐8 has good concurrent and predictive validity in different patient groups. The advantages of this survey over other assessment strategies include its low cost, simplicity, and efficiency to measure adherence. A Chinese version of the MMAS‐8 tailored‐made specifically for Hong Kong Chinese hypertensive patients was employed.

#### 2.4.1 Randomization

A unique serial number was assigned for each participant. Subsequently, the randomization process by using SCRAED was performed. A randomized list of participants was referred to the research team for receiving subsequent treatment in a 1:1 allocation ratio.

#### 2.4.2 Data Analysis

Baseline characteristics were compared between the two groups using two‐tailed Student's *t*‐tests and two‐tailed chi‐square tests for continuous and categorical variables, respectively. Student's *t*‐test was used in testing a statistical hypothesis about the mean of a normal population with an unknown variance and sample size *n* was below 30 [13]. As for the Chi‐square test, the statistical hypothesis test was used to determine whether two variables were related or not [14]. Intention‐to‐treat analysis was performed, with variables such as sex, body mass index (BMI), physical activity, education, smoking habits, and baseline adherence score included as covariates. The Last Observation Carried Forward (LOCF) method was applied for missing data. All analyses were conducted using SPSS 20.0, with *p* < 0.05 considered statistically significant [15].

## 3 Results

### 3.1 Characteristics of Participants

**Figure 1:**
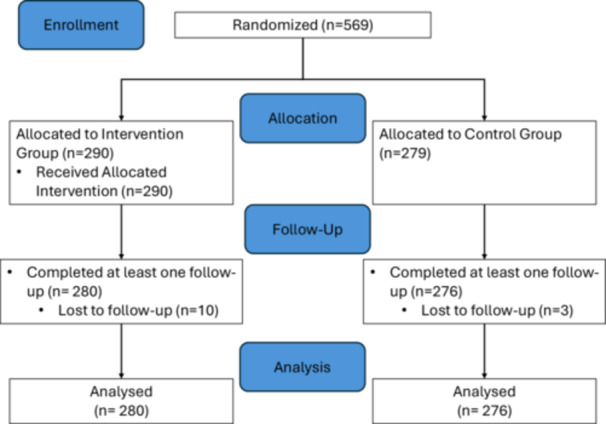
Flow diagram of the progress on the enrollment, allocation, dropout detail of this study. The figure was adapted from the CONSORT 2010 Checklist published by equator network.

Figure 1 shows the details of enrollment details in this study as referenced to the CONSORT checklist published by equator network [16]. A total of 569 patients were recruited from NGOs and community‐based centers, with 290 assigned to the intervention group and 279 to the control group. Among these, 556 completed at least one follow‐up assessment and were included in the analysis. The average age was 62.48 (±9.02) years, with no significant difference between the control and intervention groups. There was a significant difference in daily sitting time, with the control group spending an average of 7.40 h (SD 2.44) compared to 6.17 h (SD 4.74) in the intervention group. Significant differences were observed in satisfaction related to physical health, family activity, general well‐being, vision for work or hobbies, overall happiness, medication, and life quality. Differences were also noted in leisure activities and financial conditions (Table 1).

**Table 1:** Participant characteristics for continuous variables.

	Overall		Control		Intervention		*t*‐test
	Mean	Standard deviation	Mean	Standard deviation	Mean	Standard deviation	*p*‐Value
A1 Age	62.48	9.02	63.24	9.91	61.77	8.05	0.058
B1 How much time did you spend sitting every day for the last 1 week? Average of __ hours per day	6.79	3.80	7.40	2.44	6.17	4.74	**< 0.001**
C1 Consider all factors and, in the past 1 week, your satisfaction with the following conditions… (1–5)							
a. Physical health	3.39	0.76	3.53	0.63	3.26	0.84	**< 0.001**
b. Mood	3.41	0.74	3.44	0.63	3.37	0.82	0.224
c. Work	3.39	0.66	3.44	0.60	3.33	0.74	0.089
d. Family activity	3.44	0.81	3.67	0.66	3.24	0.88	**< 0.001**
e. Social relationship	3.31	0.76	3.32	0.60	3.29	0.88	0.670
f. Family relationship	3.76	0.77	3.83	0.66	3.70	0.86	0.052
g. Leisure activities	3.36	0.77	3.43	0.64	3.29	0.86	**0.033**
h. Ability to play a role in daily life	3.66	0.71	3.61	0.59	3.71	0.80	0.081
i. Sexual desire, interest, and/or performance	3.34	0.75	3.32	0.66	3.36	0.82	0.518
j. Pecuniary condition	3.51	0.70	3.58	0.60	3.44	0.78	**0.019**
k. Living situation	3.65	0.69	3.70	0.58	3.61	0.78	0.157
l. Be able to feel good health without dizziness, poor condition or fall	3.58	0.80	3.71	0.56	3.45	0.95	**< 0.001**
m. Vision for the work or hobby aspects	3.41	0.72	3.67	0.57	3.18	0.77	**< 0.001**
n. Overall happiness	3.69	0.66	3.84	0.51	3.55	0.75	**< 0.001**
o. medication	3.60	0.65	3.81	0.47	3.40	0.73	**< 0.001**
p. Overall life	3.63	0.58	3.73	0.52	3.54	0.62	**< 0.001**
C2 What do you think of your health status in the last month? (1–5)	2.70	0.91	2.74	0.88	2.66	0.94	0.326

Significant values are in bold.

### 3.2 Blood Pressure Control and MMAS‐8 Score Within Groups

No significant differences were observed in MMAS‐8 scores between groups at the 3‐month (6.52 vs. 6.71, *p* = 0.151, Cohen's *d* = −0.09) follow‐ups. However, at the 6‐month (6.53 vs. 6.93, *p* = 0.003, Cohen's *d* = −0.27) and 12‐month follow‐up (6.56 vs. 7.06, *p* < 0.001, Cohen's *d* = −0.28), the intervention group had a significantly higher MMAS‐8 score compared to the control group. For systolic blood pressure (SBP), no significant differences were found at 3 months and 6 months, but at 12 months, the intervention group's SBP was lower than control group (*p* = 0.07, Cohen's *d* = 0.18). For diastolic blood pressure (DBP), significant differences were observed at the 3‐month follow‐up, with the intervention group having a DBP of 78.86 (±9.74) compared to 77.06 (±9.37) in the control group (*p* = 0.04, Cohen's *d* = −0.10). No significant differences were noted at the 6‐ and 12‐month follow‐ups (Table 2).

**Table 2:** MMAS‐8 score and blood pressure control between control and intervention groups.

*t*‐test					
		Control group	Intervention group	*p*	Cohen's *d* a
	MMAS‐8	6.52 ± 1.51	6.71 ± 1.47	0.151	−0.09
**3 Month**	Systolic blood pressure	126.71 ± 11.37	127.32 ± 10.50	0.54	−0.04
	Diastolic blood pressure	77.06 ± 9.37	78.86 ± 9.74	**0.04**	−0.10
	MMAS‐8	6.53 ± 1.45	6.93 ± 1.26	**0.003**	−0.27
**6 Month**	Systolic blood pressure	128.25 ± 10.54	128.02 ± 10.23	0.83	0.06
	Diastolic blood pressure	77.37 ± 9.80	77.31 ± 10.11	0.95	0.15
	MMAS‐8	6.56 ± 1.44	7.06 ± 1.40	**< 0.001**	−0.28
**12 Month**	Systolic blood pressure	129.12 ± 15.79	126.76 ± 11.23	0.07	0.18
	Diastolic blood pressure	76.91 ± 9.01	76.75 ± 13.13	0.89	0.07

Significant values are in bold.

^a^ Control group as group 1; intervention group as group 2.

Chi‐square tests revealed significant differences in MMAS‐8 scores at the 12‐month follow‐up, with 68.6% (192) of the intervention group achieving optimal results compared to 57.6% (159) in the control group (*p *= 0.007). Although no significant differences were observed in SBP at 3 (*p *= 0.752), 6 (*p *= 0.908), and 12 (*p *= 0.100) months, 18.1% (50) of the intervention group reported optimal SBP control at 12 months compared to 13.0% (36) of the control group. For DBP, significant differences were only observed at 3 months, with the control group showing better results (59.4% vs. 49.3%, *p *= 0.017). No significant differences were observed at 6 and 12 months (Table 3).

**Table 3:** Proportion of optimal MMAS‐8 score and blood pressure control between control and intervention groups.

Chi‐Square test						
			Control group	Intervention group	Total	*p*
MMAS‐8 (Month 3)	Optimal^a^	Count	148	165	313	=0.207
		% within GP	53.6%	58.9%	56.3%	
	Abnormal	Count	128	115	243	
		% within GP	46.4%	41.1%	43.7%	
MMAS‐8 (Month 6)	Optimal^a^	Count	158	171	329	=0.359
		% within GP	57.2%	61.1%	59.2%	
	Abnormal	Count	118	109	227	
		% within GP	42.8%	38.9%	40.8%	
MMAS‐8 (Month 12)	Optimal^a^	Count	159	192	351	**=0.007**
		% within GP	57.6%	68.6%	63.1%	
	Abnormal	Count	117	88	205	
		% within GP	42.4%	31.4%	36.9%	
Systolic blood pressure (Month 3)	Optimal^b^	Count	58	55	113	=0.752
		% within GP	21.0%	19.9%	20.5%	
	Abnormal	Count	218	221	439	
		% within GP	79.0%	80.1%	79.5%	
Systolic blood pressure (Month 6)	Optimal^b^	Count	44	45	89	=0.908
		% within GP	15.9%	16.3%	16.1%	
	Abnormal	Count	232	231	463	
		% within GP	84.1%	83.7%	83.9%	
Systolic blood pressure (Month 12)	Optimal^b^	Count	36	50	86	=0.100
		% within GP	13.0%	18.1%	15.6%	
	Abnormal	Count	240	226	466	
		% within GP	87.0%	81.9%	84.4%	
Diastolic blood pressure (Month 3)	Optimal^c^	Count	164	136	300	**=0.017**
		% within GP	59.4%	49.3%	54.3%	
	Abnormal	Count	112	140	252	
		% within GP	40.6%	50.7%	45.7%	
Diastolic blood pressure (Month 6)	Optimal^c^	Count	145	132	277	=0.268
		% within GP	52.5%	47.8%	50.2%	
	Abnormal	Count	131	144	275	
		% within GP	47.5%	52.2%	49.8%	
Diastolic blood pressure (Month 12)	Optimal^c^	Count	147	132	279	=0.202
		% within GP	53.3%	47.8%	50.5%	
	Abnormal	Count	129	144	273	
		% within GP	46.7%	52.2%	49.%	

*Note:* Bold values are statistically significant.

^a^ Optimal: MMAS‐8 Score > 6.

^b^ Optimal: Systolic blood pressure < 120 mmHg.

^c^ Optimal: Diastolic blood pressure < 80 mmHg.

## 4 Discussion

Our results observed that patients who used the “**My eDrug Manager**” presented with higher drug adherence than that in the control group. This implied that using “**My eDrug Manager**” as a patient empowerment tool might help to achieve optimal control of blood pressure among users. This also suggested that the “**My eDrug Manager**” electronic application tool was useful in improving users' drug adherence. The results from this study aligned with previous related studies. For instance, one study reported that drug adherence improved from 65% at baseline to 94% after adopting a telemedicine chronic disease management programme [17]. Also, there is one clinical trial investigated an adherence app called CollaboRhythm for patients with hypertension. It allowed tracking of medications and receiving recommendations and reminders. After 3 months, the participants using app had a greater decrease (~10 mmHg) in blood pressure than the control group [18]. Another recent RCT evaluating the effectiveness of Medisafe app on drug adherence in hypertensive older adults was published in 2018. Patients in the treatment group were instructed to setup this app that incorporated reminder alerts, adherence reports, and peer communication support. A significant improvement in change of drug adherence from baseline to 3 months was found after intervention [15].

It is interesting to note that MMAS‐8 score reported no significance differences at month 3 and 6 follow‐up, but significance differences were reported at month 12. This finding is similar to previous studies which pointed out that high drug adherence could not be observed in a short timeframe. In other words, drug adherence might not be easily optimized in a short‐term period. This also applies to blood pressure control. Hence, higher drug adherence and better blood pressure control might be observed if the study was extended to capture long‐term outcomes [19]. Future studies could be focused on the long‐term drug adherence and management of hypertension among senior participants. In addition, the patient perception on the application also awaited to be investigated for providing a more user‐friendly design and increase the utility of the application.

### 4.1 Limitations

This study also had some limitations. As the level of medication adherence was measured by the eight‐item self‐administered Morisky Medication Assessment Scale (MMAS‐8 score), which is based on patients' self‐perception, bias might be inevitable. In addition, the daily sitting time between control and intervention group shown significant difference. The baselines imbalance may affect the blood pressure outcomes found in this study. Future studies should be focused on balancing groups concerning sitting time to better understand its role in moderating intervention effects. Furthermore, the longer period of follow up, such as 3‐, 5‐, and 10‐years follow up were recommended to provide more comprehensive results based on the findings from this study. Despite a difference between control and intervention group was observed in this study, the difference may not represent a meaningful improvement in adherence.

## 5 Conclusions

“My eDrug Manager” mobile app has potentially improved the level of medication adherence, which might bear potential to enhance optimal control of blood pressure among older adults with hypertension in a longer‐term follow‐up period. The findings of this study could inform the benefits and room for improvement of the eHealth apps and its relevant Health Management Module.

## Author Contributions

**Martin C. S. Wong:** conceptualization, supervision, writing – review and editing. **Claire Chenwen Zhong:** conceptualization, supervision, writing – original draft; data curation, formal analysis. **Siu Hin Wong:** writing – original draft, data curation, formal analysis. **Chung Yi Lo:** writing – original draft, data curation, formal analysis. **Man Kin Yim:** writing – original draft. **Junjie Huang:** writing – original draft, conceptualization, supervision, formal analysis, data curation.

## Ethics Statement

This study was approved by the Joint CUHK‐NTEC Clinical Research Ethics Committee (CREC Ref. no: 2018.650).

## Consent

The authors have nothing to report.

## Conflicts of Interest

The authors declare no conflicts of interest.

## Transparency Statement

The lead authors Martin CS Wong, Claire Chenwen Zhong, and Junjie Huang affirm that this manuscript is an honest, accurate, and transparent account of the study being reported; that no important aspects of the study have been omitted; and that any discrepancies from the study as planned (and, if relevant, registered) have been explained.

## Data Availability

The data that support the findings of this study are available from the corresponding author upon reasonable request. The datasets used and/or analysed during the current study are available from the corresponding author on reasonable request.
